# Detecting delirium in nursing home residents using the Informant Assessment of Geriatric Delirium (I-AGeD): a validation pilot study

**DOI:** 10.1007/s41999-022-00612-w

**Published:** 2022-02-10

**Authors:** Pia Urfer Dettwiler, Franziska Zúñiga, Stefanie Bachnick, Beatrice Gehri, Jos F. M. de Jonghe, Wolfgang Hasemann

**Affiliations:** 1 Nursing Home Frenkenbündten, Gitterlistrasse 10, 4410 Liestal, Switzerland; 2 Institute of Nursing Science, University of Basel, Bernoullistrasse, 4056 Basel, Switzerland; 3 University Psychiatric Clinics, Wilhelm Klein-Strasse 27, 4002 Basel, Switzerland; 4 Private Practice, DC VerzuimDiagnostiek , Tesselschadestraat 4, 1054 ET msterdam, The Netherlands; 5 University Department of Geriatric Medicine FELIX PLATTER, Burgfelderstrasse 101, 4002 Basel, Switzerland

**Keywords:** Nursing homes, Delirium, Delirium assessment, Aged, Validation study

## Abstract

**Purpose:**

Early delirium detection in nursing home residents is vital to prevent adverse outcomes. Despite the potential of structured delirium screening tools to enhance delirium detection, they are rarely used in nursing homes. To promote delirium screening tools in nursing homes, they should be easy to integrate into the daily routine of care workers. The I-AGeD, was developed as a simple and easily understandable tool to detect delirium in older adults. The aims of this study were to record the prevalence of delirium, to investigate the feasibility of the I-AGeD, and to compare these results with the DSM-5 as the reference standard.

**Methods:**

This is a cross-sectional prospective single-center pilot study. Seven registered nurses assessed the participants with the I-AGeD. The research assistant conducted delirium assessments based on the DSM-5 criteria, to identify delirium symptoms for the same participants. The feasibility test was verified using a five-point Likert scale ranging from very easy to very difficult.

**Results:**

85 nursing home residents participated in the study. A delirium prevalence of 5.9% was found. The sensitivity was 60% and specificity 94% at a cut point of ≥ 4 to indicate delirium. The feasibility test showed that the 10 items of the I-AGeD were easy or very easy to answer.

**Conclusion:**

The I-AGeD showed an acceptable performance to assess delirium in nursing home residents. Additionally, it was found feasible and due to its brevity the I-AGeD could easily be integrated into the routine of daily care in nursing homes.

## Key summary points

**Aim:**

The aims of this study were to record the prevalence of delirium in residents with the DSM-5, to investigate the feasibility of the I-AGeD in a Swiss nursing home and to compare the results of the I-AGeD with the DSM-5 criteria as the reference standard.

**Findings:**

This study shows that the I-AGeD is suitable for detecting delirium in nursing home residents with a sensitivity of 60% and a specificity of 94%.

**Message:**

The I-AGeD is a simple, brief and feasible assessment tool for the detection of delirium in nursing home residents.

## Background

Delirium is a common and acute disorder of cognition in the elderly characterized by inattention, changes in consciousness and cognitive function and fluctuating symptoms over the course of the day [1]. Based on these symptoms, delirium diagnosis is internationally defined according to the criteria of the Diagnostic and Statistical Manual Version 5 (DSM-5) [1]. Delirium in elderly people occurs in all health care settings, thus also in nursing homes with their elderly population [2]. The prevalence of delirium in nursing home residents ranges from 1.4 to 70% [3–6].

Although delirium in nursing home residents is very common, care workers often fail to recognize it [2, 6]. A study from Canada, with nursing home residents with and without a cognitive impairment shows that only 25–67% of the residents with delirium were identified [6]. The fluctuating course of both the hyperactive and the hypoactive form as well as the varying manifestations of delirium symptoms make it difficult to detect delirium [7]. The hypoactive form in particular is often overlooked by care workers in nursing homes, as the symptoms, such as inactivity and drowsiness, are not recognized as delirium symptoms [2, 8].

However, early delirium detection in nursing home residents is vital. It may help in avoiding adverse outcomes such as higher mortality, hospitalization, functional and cognitive decline [2, 9]. Undetected delirium is also highly linked with increased health care costs and a higher demand of care workers [10, 11]. Furthermore, delirium is associated with patient safety issues such as falls and pressure ulcers [9]. Because of all these risks and connected complications, delirium can be very stressful for nursing home residents and their relatives [12]. Although studies only exist on hospitalized patients and not on nursing home residents, the resulting high workload and increased burden for care workers experienced by nursing homes caring for delirious patients may be comparable [13].

To improve delirium management enhanced delirium detection with structured assessment tools in routine care would be helpful [6]. A wide range of delirium assessment tools have already been developed, but only five of them have been translated into German: The Confusion Assessment Methods (CAM) and its operationalized version of the original CAM algorithm the modified Confusion Assessment Method for the Emergency Department (mCAM-ED), the Delirium Observation Scale (DOSS), the Confusion Scale developed by Champagne and Neelon (NEECHAM) and the 4A’s Test (4AT) [14–18]. Despite their known potential in detecting delirium, in general delirium assessment tools are rarely used in nursing homes [6]. This is due to lower nursing qualification levels among the care workers and a lack of training as well as time pressure, which results from a lack of registered nurses in nursing homes [19]. Because daily care of residents is mainly provided by care workers with lower qualification levels [20, 21], delirium assessment tools in nursing homes must be easy to use and to integrate into the daily routine of all care workers [6, 20, 22]. In current literature, there is no mention of a delirium assessment tool that can be used by all qualification levels of care, although this would be needed in the nursing home context. However, two tools already exist for the use by lay persons: The Informant Assessment of Geriatric Delirium **(**I-AGeD) and the Family Confusion Assessment Method (FAM-CAM) [23, 24]. The FAM-CAM does not contain any lay terms and requires previous training for the application [25]. In comparison, the I-AGeD was developed for lay persons without previous training as a simple and easily understandable questionnaire to detect delirium in the elderly [23].

The only study carried out using I-AGeD focused particularly on delirium in elderly hospital patients with and without dementia [23]. So far, no study has ever validated the I-AGeD in the context of nursing home residents. Since the I-AGeD is a brief, simple and validated tool for detecting delirium in the hospitalized elderly and especially developed for family members and informal caregivers, it lends itself to being used by care workers of all qualification levels in nursing homes.

As the presentation of delirium symptoms in long-term care facilities may differ from presentations in hospitals, for this paper a validation study has been carried out to investigate the usefulness of the I-AGeD in this setting. To reduce observer bias, registered nurses (RNs) are used to rate the I-AGeD. The professional training of RNs results in high compliance with tasks, which will allow us to reduce noise in the data. In a second step, a reliability study will be performed to investigate the performance of the I-AGeD by care workers of different qualification levels. Accordingly, the specific aims of this study are: (1) to describe the prevalence of delirium in residents as assessed with DSM-5 reference standard used by the research assistant, (2) to investigate the feasibility of the I-AGeD in a Swiss nursing home, and (3) to compare the results of the I-AGeD with the DSM-5 criteria as the reference standard.

## Methods

### Design

This is a cross-sectional single-center pilot study. In this pilot study the validation, feasibility and the interrater reliability of the I-AGeD have been tested.

### Setting and participants

One nursing home in the German speaking part of Switzerland in the canton Basel-Landschaft participated in this study. It is a 134-bed nursing home with six wards. A consecutive sample of nursing home residents, with and without cognitive impairments, was included.

Participants in the study were nursing home residents of 60 years or older who had a Cognitive Performance Scale (CPS) score between 0 and 5 (intact to severe impairment) out of 6 [26]. Those residents who were unable to communicate verbally (e.g. aphasia, coma), or were nearing the end of their life were excluded from this study.

### Variables and measurements

#### Measurements

Demographical data such as age (years), gender (male, female), the existence of a diagnosed dementia or a suspected dementia and duration of stay since admission (days) were collected from the health records. The level of cognitive impairment was determined based on the CPS. The CPS score ranges from 0 (intact) to 6 (very severe impairment) [26]. In Switzerland, the level of care dependency is measured on a scale of 1–12. Each of these 12 levels stands for a time unit of care of 20 min per day. Level 1 contains up to 20 min and level 12 more than 220 min of daily care [27].

#### I-AGeD

The I-AGeD is a questionnaire for family members to detect delirium in elderly patients [23] (Appendix A). Thus far only one study which validated the I-AGeD exists. This study used the reference standard DSM-IV, a previous version of today’s standard, DSM-5, for the diagnosis of delirium [23]. It was validated with a sensitivity 77.4% and a specificity 63.2% with a cut-off of greater than 4 to indicate delirium [23]. In patients without dementia the sensitivity was 100% and the specificity 65.2% [23].

In this study the I-AGeD was validated in the nursing home setting. RNs from each of the six wards rated delirium in nursing home residents with the following ten questions of the I-AGeD [23]: *(1) “I do not recognize him/her as their usual self”, (2) “I often have to repeat things to get his/her attention”, (3) “He/she is less alert and/or appears to be drowsy during the daytime”, (4) “He/she has little spontaneous movement and hardly moves the upper limbs”, (5) “He/she is often awake at night and sleepy during the day”, (6) “He/she has recently become more forgetful”, (7) “When the conversation stops, his/her eyes close”, (8) “He/she is difficult to awaken”, (9) “He/she is combative and struggles to get free”,* and *(10) “He/she says strange things that don`t make any sense”*. The I-AGeD contains dichotomous “yes” and “no” answers. The presence of delirium was affirmed, if four or more questions were answered with “yes”. In this study a cut-off of four or more was used to indicate delirium according to the Swiss guideline for delirium management in nursing homes [28], whereas the original cut-off used by Rhodius et al. [23] is five or more.

#### DSM-5

The criteria of DSM-5 are internationally acknowledged as the reference standard of delirium [29] and in this study, they were used by the research assistant (RA) to diagnose delirium in the nursing home residents (Appendix B). According to DSM-5 delirium is defined with the following five criteria [1]:

- Criterion A: Disturbance in attention and awareness
- Criterion B: Acute change from baseline attention and awareness with fluctuations over the day
- Criterion C: An additional disturbance in cognition
- Criterion D: The disturbance in Criteria A and C are not better explained by a neurocognitive disorder or coma
- Criterion E: Evidence that disturbance is a consequence of another medical condition, substance intoxication or withdrawal, exposure to a toxin or multiple etiologies

In this study, two sources were used to rate the five criteria of the DSM-5: structured interviews with the mCAM-ED (Criteria A-D) and additional information from the health record (Criterion E).

#### mCAM-ED

The mCAM-ED [30] is an operationalized version of the original CAM algorithm [31] (Appendix C and D). It has been validated in the Emergency Department of a Swiss University Hospital and was used to detect delirium in older people [30]. As the mCAM-ED was also used intensively by the consultation service of the University Hospital in medical and surgical departments, this approach was considered as appropriate to use as an assessment tool in the nursing home. These patients were included in the study sample of Hasemann et al. [25, 30] and were also screened using the mCAM-ED [30]. It consists of the month of the year backwards test (MOTYB) [32], the Mental Status Questionnaire (MSQ) [33], the Comprehension Test [34] as well as the evaluation of an acute onset of symptoms, fluctuation over the course of the day and altered level of consciousness, as measured by the Modified Richmond Agitation and Sedation Scale (mRASS) in our study [35] (Appendix E).

#### Evaluation of mCAM-ED

According to the CAM algorithm, delirium may be present when either ((1a acute changes in cognition (MSQ; MOTYB) OR 1b fluctuation course (observation)) AND 2 the presence of inattention (MOTYB) AND (3 disorganized thinking (Comprehension Test) OR 4 an altered level of consciousness (mRASS)), i.e. (1a OR 1b) AND 2 AND (3 OR 4) are shown [30].

#### Feasibility test

The feasibility of each item of the I-AGeD was verified using a five-point Likert scale ranging from very easy to very difficult. The feasibility test provides information on how difficult it was to assess delirium in each included resident. In an open text field, the RNs could add more information about the feasibility of the I-AGeD.

### Data collection

The data was collected from September 2019 until October 2019. A total of seven RNs performed the data collection for this study. The seven RNs have the same level of nursing qualification but different work experience which ranges from 2 to 12 years. In five of the six wards one RN assessed the participants with the I-AGeD. The RA conducted delirium assessments based on the DSM-5 criteria as the reference standard, to capture delirium symptoms for the same participants within 1 h of the RN even in residents with suspected delirium. This guaranteed that all residents received the same formal procedure to diagnose delirium. The RA was blinded to the ratings of the I-AGeD, in order to collect the data without bias. Unclear delirium diagnoses were discussed with the principal investigator (PI). Furthermore, each resident with delirium was discussed with the PI who was blinded to all ratings.

#### Training of RNs and RA

The seven RNs underwent a 1-day training by the PI. The training included theoretical inputs on delirium and information on how to administer the I-AGeD. The RA, who was an advanced practice nurse in geriatrics with 5 years of experience in this role, received a 5-day training also provided by the PI. This included theoretical inputs on delirium and the application of the mCAM-ED and DSM-5 criteria. The RA applied the mCAM-ED in the Emergency Department, on the wards of geriatric patients in the University Hospital Basel and in the participating nursing home and was supervised by the PI.

### Ethical considerations

This study was approved by the ethics committee of “Nordwest- und Zentralschweiz” (Project ID 2019-01184). A written informed consent was obtained for each eligible nursing home resident. A proxy informed consent was obtained by family members or legal advisors, for residents with a CPS score of 4 and 5.

### Data analyses

The collected data was analyzed with descriptive statistics. Categorical data was processed according to frequencies and percentages, continuous variables according to means, standard deviations and ranges. In the case of skewed data, the median and first and third interquartile ranges (Q1, Q3) were computed. All demographic variables have been compared between nursing home residents with and without delirium. The prevalence, as the first aim of this study, was obtained with descriptive data. These analyses were performed with IBM SPSS (Statistical package for the Social Sciences) version 25.0. To answer aim two, the feasibility test was analyzed with percentages, means, standard deviations and ranges. The open text fields were analyzed and interpreted using the thematic analysis according to Braun and Clarke [36]. This was carried out by conducting the codebook analysis through building domain summaries [37]. For aim three concerning the performance of the I-AGeD and DSM-5, the sensitivity and specificity as well as the positive- and negative- predictive value (PPV and NPV) were estimated and presented together with the two-sided 95% confidence intervals (CI). The exact CIs were calculated according to the method of Collett [38]. Additionally, positive and negative likelihood ratios (LR+ and LR−) were computed and presented with their two-sided 95% CI, calculated according to the method of Simel, Samsa & Matchar [39]. These analyses were conducted using R Version 3.5.3.

## Results

### Descriptive characteristics

**Fig. 1:**
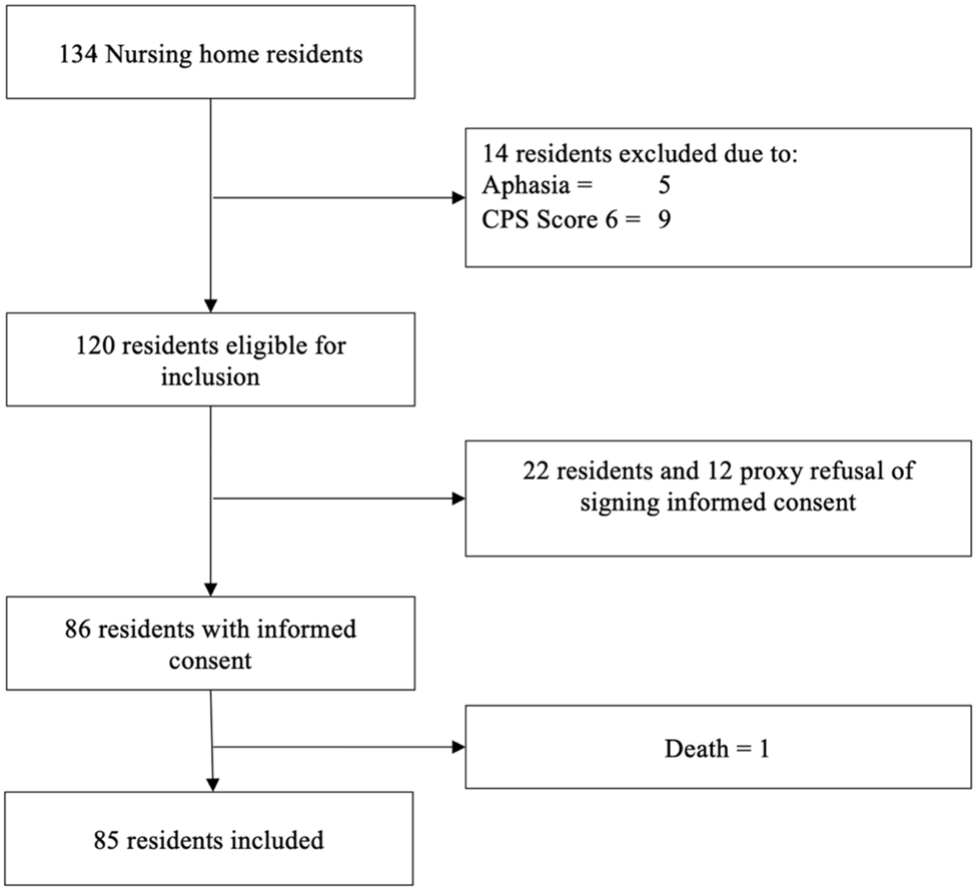
Flow of participants

Of a total of 134 nursing home residents, 14 were excluded (aphasia or a CPS score of 6). This resulted in 120 residents who were eligible to participate in this study. The informed consent was not obtained from 22 residents and 12 proxy who refused to sign it. This yielded 86 residents with informed consent. During the data collection one resident died. Finally, 85 residents were included in this study (Fig. 1). The included nursing home residents had a mean age of 85.5 (SD 7.6 years) and 64.7% were female. The health records of 41.2% contained documentation of dementia. The median CPS score of the included residents was 2 (mild impairment) (*Q*1 = 1.0; *Q*3 = 3.0*)*. The median level of care dependency was 5 (81–100 min of daily care) (*Q*1 = 3.0; *Q*3 = 7.0*)*, and the median of days since admission was 746 days (*Q*1 = 335.0; *Q*3 = 1192.8) (Table 1).

**Table 1:** Nursing home residents characteristics (*n* = 85)

Characteristics	Total sample	Delirium	No Delirium
*N* (%)	85 (100)	5 (5.9)	80 (94.1)
Age*			
Mean (SD)	85.5 (7.5)	92.8 (3.4)	85.1 (7.5)
Gender			
Female, *N* (%)	55 (64.7)	5 (100)	50 (62.5)
Male, *N* (%)	30 (35.3)	0 (0)	30 (37.5)
Neurocognitive impairment			
Dementia, *N* (%)	35 (41.2)	2 (40.0)	33 (41.3)
No dementia, *N* (%)	50 (58.8)	3 (60.0)	47 (58.8)
CPS			
Median (*Q*1; *Q*3)	2 (1.0; 3.0)	3 (2.5; 3.0)	2 (1.0; 3.0)
Levels of care dependency			
Median (*Q*1; *Q*3)	5 (3.0; 7.0)	7 (5.5; 8.0)	5 (3.0; 7.0)
Days since admission			
Median (*Q*1; *Q*3)	746 (335.0; 1192.7)	1135 (373.0; 3516.5)	840.5 (335.0; 1411.3)

*SD* standard deviation; *CPS* Cognitive Performance Scale, *Q1; Q3* first and third quartile

*Significant *p* < 0.05

Nursing home residents with and without delirium did not differ in most demographic characteristics with the exception of age. Residents with delirium were significantly older than residents without delirium (*p* = 0.011) (Table 1). According to the DSM-5 reference standard a delirium prevalence of 5.9% was found during the data collection.

### Feasibility test

For the sample of 85 assessments, six RNs completed the feasibility test. The mean difficulty of each of the ten items of the I-AGeD was rated using a five-point Likert scale from 1 (very easy) to 5 (very difficult). The result showed mean ratings ranging between 1.2 and 1.5, meaning that overall the items were easy or very easy to answer according to the respondents. None of the RNs rated any item in any of the situations contained in the I-AGeD as “very difficult” to answer.

Approximately 10% of the open text field of the feasibility test were filled out by the RNs. The thematic analysis revealed three domain summaries, that challenge delirium assessment: “residents with dementia”, “residents with hearing impairment” and “care workers` unfamiliarity with residents”. RNs have difficulties in assessing nursing home residents with dementia using the I-AGeD, since RNs have problems to differentiate between symptoms of dementia and delirium or a combination of dementia and delirium. The second domain summary “residents with hearing impairment” describes the RNs` difficulty in assessing nursing home residents who have hearing problems. Such residents often do not understand questions acoustically and can therefore not answer the question or give an inadequate answer. In such cases it is difficult for the RN to interpret inadequate answers as a consequence of hearing impairment, dementia or delirium. The third domain summary is “unfamiliarity with residents” and refers to cases where the RN does not know the resident well. The RN must then consult the health records and read the nursing reports or occasionally needs information from another RN who knows the resident better.

### Performance

The comparison of the RNs using the I-AGeD and the RA assessing nursing home residents with the DSM-5 are shown in Table 2. As per reference standard DSM-5, the I-AGeD correctly identified with a sample of *n* = 85, 3 out of 5 (60%) (95% CI 0.15, 0.95) of all nursing home residents with delirium as being delirious when screened by the RA (sensitivity). For the 80 non-delirious residents, 75 (94%) were correctly identified with the I-AGeD (95% CI 0.86, 0.98) as being non-delirious in comparison to the reference standard (specificity). Amongst nursing home residents who were screened positive, 38% were truly positive (95% CI 0.09, 0.76) (positive predictive value). Whereas 97% of those who were screened as non-delirious were truly negative (95% CI 0.91, 1.00) (negative predictive value). It was revealed, that the probability of a nursing home resident being correctly classified as delirious with the I-AGeD was 9.6 (95% CI 3.16, 29.13) times higher than being incorrectly classified (positive likelihood ratio). However, the probability of misclassifying a delirious nursing home resident as non-delirious with the I-AGeD was 43 times (95% CI 0.15, 1.25) lower than judging a non-delirious nursing home resident as non-delirious (negative likelihood ratio) (Table 3).

**Table 2:** Cross-table of I-AGeD results by DSM-5 delirium detection rate (*N* = 85)

I-AGeD	DSM-5	
	Delirium	No delirium
Delirium	3	5
No delirium	2	75

*I-AGeD* Informed Assessment of Geriatric Delirium, *DSM-5* Diagnostic and Statistical Manual of Mental Disorders, Fifth Edition = Delirium diagnosis

**Table 3:** Performance of the I-AGeD versus DSM-5 (*N* = 85)

	*N* = 85	
	Estimate	95% CI
Sensitivity	0.60	0.15, 0.95
Specificity	0.94	0.86, 0.98
Positive predictive value	0.38	0.09, 0.76
Negative predictive value	0.97	0.91, 1.00
Positive likelihood ratio	9.60	3.16, 29.13
Negative likelihood ratio	0.43	0.15, 1.25

*CI* confidence interval

## Discussion

Our study demonstrated an acceptable performance in assessing delirium in nursing home residents with the I-AGeD. The results demonstrate that RNs identified non-delirious residents with the I-AGeD with an excellent specificity, i.e. a good positive likelihood ratio, while delirious residents were recognized with a moderate sensitivity, corresponding with a weak negative likelihood ratio.

The I-AGeD achieved an excellent specificity in this setting with a high number of residents with dementia in a nursing home setting. This is very important, as a high specificity results in a low false positive rate. With fewer false positive results, the demand for further assessment is lower, resulting in a reduced workload for care workers. Comparison with the results in Rhodius et al. [23], shows that they achieved a lower specificity. The high specificity of our study is remarkable because we adjusted the cut-off value from five to four points. With a lower cut-off we should have expected a deterioration of the specificity, but this was not the case. One explanation of the resulting high specificity might be, that we used qualified RNs instead of lay persons as in the study of Rhodius et al. [23] to rate delirium in nursing home residents. We will further explore how care workers with Federal Diplomas/Certificates of Vocational Education and Training and nursing assistants would rate the residents with the I-AGeD in a follow-up study.

The sensitivity was slightly lower than the original developed scale of Rhodius et al. [23]. Although the cut-off was lower in comparison to the original study, we did not achieve a higher sensitivity. It has to be acknowledged that two out of five residents in our sample had dementia. In the context of dementia, delirium superimposed on dementia is often overlooked [40]. It should be mentioned that, with a false positive rate of 6% the performance of the I-AGeD in our study was excellent. In the study of Teale, Munyombwe, Schuurmans, Siddiqi, and Young [41] the false positive rate in a nursing home sample with 50% cognitively impaired residents, was with 29% higher compared to when the DOSS was used.

The modest sensitivity shows that the RNs have not recognized all delirious residents, which is consistent with Voyer et al. [6] who had a sensitivity of 51% comparing the CAM with RN and RA in nursing home residents. They assumed that RNs, with a work experience in geriatric care of 9 years or less, have more difficulties in recognizing delirious residents than RNs with longer work experience. The work experience of the participating RNs in our study ranged between 2 and 12 years, although only one RN in the study had more than 9 years working experience. Another reason for not detecting delirium might be related to assessing it by means of observation only (I-AGeD) rather than by structured interview in order to rate the criteria of DSM-5, as it was conducted by the RA. These findings are also in accordance with the results described by Voyer et al. [6], where RNs did not recognize delirium symptoms in residents as successfully as the RA, who used structured delirium assessment tools. The lack of expertise may be another reason for the modest sensitivity. The RA attended a 5-day training program conducted by the PI, which also included practical training. In contrast, the RNs only had 1 day of training and no mentoring. Several studies indicate that training and mentoring yield increasingly better rates of detecting delirious residents [6, 42]. These studies show clearly that care workers should be given more theoretical and practical training, to improve their competence in delirium detection [6].

According to the feasibility test, the RNs rated the I-AGeD as easy to very easy to answer. These findings show a high acceptability of the I-AGeD for RNs working in nursing homes. In only ten delirium assessments, four RNs mentioned challenges in the use of the I-AGeD. This occurred mainly when residents had dementia and the distinction between delirium and dementia proved to be difficult. This is in line with the study of Morandi and Bellelli [40], who mention the difficulty in recognizing delirium in patients with severe dementia. Another domain summary which emerged from the thematic analysis, was assessing residents with hearing impairment. Hearing impairment is a known risk factor for the development of delirium. Also, it may hamper delirium assessment as misleading answers from residents may be mistaken by the interviewer as delirium [43]. For this reason, it is important to check in advance whether the hearing aid is functional. Inouye [44] solved this problem by using additional hearing amplifiers, which were used on patients with severe hearing impairments. Finally, the third domain summary is “unfamiliarity with residents”. In such a case, the third party of medical records is required to answer questions one and six of the I-AGeD.

The I-AGeD is a short and easy to use assessment instrument which takes approximately 2 min to fill out. Although it was specifically developed for lay persons to detect delirium, we believe that training is still needed before using it. When the I-AGeD was validated in the Emergency Department (ED) setting with family members, the authors did not give any formal training concerning the I-AGeD. This may have led to a low sensitivity of the I-AGeD in the ED according to a forthcoming publication of Hasemann et al. [48]. This finding is not consistent with the study by Bellelli et al. [16]. The authors refer to evidence demonstrating that formal training is not required to achieve reliable results in the detection of delirium using the 4`A`s Test (4AT), which is considered to be a brief and simple test. This statement is challenged by Myrstad et al. [45] as in their quality improvement study, sensitivity and specificity of the 4AT were shown to be insufficient. The authors concluded that formal training would have been necessary for better performance. A similar statement was made by Shulman et al. [46], that formal training is not required for delirium detection using the Sour Seven Questionnaire. The authors developed a tool for delirium detection in hospitalized patients especially for use by lay persons and untrained care workers. However, as the items are not described in lay persons` language, the results may be questioned. Additionally, a screening bias may be inherent, as all reference assessments of delirium were not consistently done by a geriatrician, but only CAM positives. Fick et al. [47] also share the opinion that no prior training is needed to recognize a delirium using the very brief Two-Step Delirium Detection Protocol which was conducted by certified nursing assistants. This protocol includes the following two items: the MOTYB and the question about the day of the week [47]. The drawback of this Two-Step Delirium Detection Protocol is that people with dementia are not able to answer these two items [48]. Since approximately 65% of nursing home residents in Switzerland are either diagnosed with or suspected to have dementia [49], this Protocol does not seem suitable for detecting delirium in residents in nursing homes by untrained care workers. We believe that distinguishing delirium from dementia needs prior training in assessing nursing home residents.

## Limitations

The main limitation of the present study lies in its small sample size. Therefore, to avoid overestimating the test performance, interpretations of the results will require special caution. Furthermore, only 41.2% of residents had an officially diagnosed dementia, although CPS values suggested a higher rate of residents with dementia. This may limit generalizability.

The approach utilized by the RA to capture delirium symptoms in residents within 1 h of the RN could not be carried out for seven residents (8%). This was due to the fact that these residents took part in group activities. They were independent and cognitively intact residents without delirium. In order to capture delirium symptoms within 1 h of the RN, in a subsequent study, residents should be informed prior to data collection of the time frame within which the assessments will be carried out.

For this study, we were interested in investigating the performance of the I-AGeD in the new setting of long-term care. We chose RNs as raters as they are the primary care persons.

## Conclusion

In this study the I-AGeD shows a good performance in nursing home residents. The focus regarding the performance of the I-AGeD was on the validity with RNs. Since the I-AGeD is a brief and simple to use assessment instrument, written in layman’s terms, further validation studies should be conducted with a larger sample and with care workers with Federal Diplomas/Certificates of Vocational Education and Training and nursing assistants. As the daily care of nursing home residents is mainly provided by them, they play an integral role in the identification of residents with delirium. The brevity of the I-AGeD shows that it can be easily integrated into daily care, allowing detection of delirium in nursing home residents. Furthermore, the I-AGeD was found to be feasible by the RNs. Our study shows that this tool could play a key role in the detection of delirium in nursing home residents, which in turn could have a positive impact on the quality of life of the residents. Training in the use of the I-AGeD and an algorithm to apply it, should be considered before implementing the I-AGeD.

## Data Availability

On request data will be provided.

## Appendix A

Informed Assessment of Geriatric Delirium (I-AGeD).

## Appendix B

Diagnostic and Statistical Manual of Mental Disorders, Fifth Edition: Delirium diagnosis criteria (DSM-5).

## Appendix C

The modified Confusion Assessment Method for the Emergency Department (mCAM-ED).

## Appendix D

Overview of the variables of the mCAM-ED.

**Table:**

Variable	Description	Measurement
MOTYB	The MOTYB evaluates the presence of inattention [50] Recite the month of the year backwards beginning with December	Every omission is an error and scored with one point More than 30 s for task, one additional point Inattention is present with a score of ≥ 3 [36]
MSQ	Screener with 10 items for cognitive impairment to test the time orientation, person, place and memory [37] Adaptation of the first and second questions of the original questionnaire, because of different setting: *(1) “what is the name of this place?” and (2) “where is this located (address)?”* have been changed to *(1) “What is the name of this nursing home?” and (2) “Where is this nursing home located?”* The remaining questions are for example: *“What is today`s date?”, “How old are you?”, “Who is the President of the United States?”*	Dichotomous questionnaire with the answers “correct”, “incorrect” or “not applicable” The MSQ score counts the number of correct answers A test score of 7 or less presents cognitive impairment
The Comprehension Test	The test evaluates disorganized or incoherent thinking [38] It contains four questions such as: *“Will a stone float on water?”* or *“Can you use a hammer to pound a nail?”*	Dichotomous questions answerable with “correct” or “incorrect” Difficulties in logical reasoning is present with a score of 2 or less
Onset of cognitive alteration and fluctuation course	Cognitive changes are rated based on observation, described in health records and information by care workers or relatives	Dichotomous question with “yes” or “no”
mRASS	Observational instrument to assess levels of consciousness [39] Hyperactive and hypoactive levels of consciousness are captured	The scores range from − 5 unarousable, 0 alert to + 4 combative Every score other than 0 indicates an altered level of consciousness

*MOTYB* month of the year backwards test; *MSQ* Mental Status Questionnaire, *mRASS* Modified Richmond Agitation and Sedation Scale

## Appendix E

The modified Richmond Agitation and Sedation Scale (mRASS).

## Code availability

IBM SPSS version 25.0, R version 3.5.3.
